# Supplementary material for: Enhanced feature matching in single-cell proteomics characterizes IFN-γ response and co-existence of cell states
Source: Nat Commun. 2024 Sep 26;15:8262. doi: 10.1038/s41467-024-52605-x (PMC11427561; doi:10.1038/s41467-024-52605-x)
Supplement: Supplementary file 1 — Supplementary Information [file 41467_2024_52605_MOESM1_ESM.pdf]

Supplementary Information

Enhanced feature matching in single-cell proteomics characterizes IFN-γ response and co-existence of cell states

Karl K. Krull[1,2,3], Syed Azmal Ali[1], Jeroen Krijgsveld[1,3,*]

[1] German Cancer Research Center (DKFZ), Heidelberg, Proteomics of Stem Cells and Cancer, Germany
[2] Heidelberg University, Faculty of Biosciences, Heidelberg, Germany
[3] Heidelberg University, Medical Faculty, Heidelberg, Germany

* Correspondence to J.K.,
email: j.krijgsveld@dkfz.de,
phone: +49-6221-421720

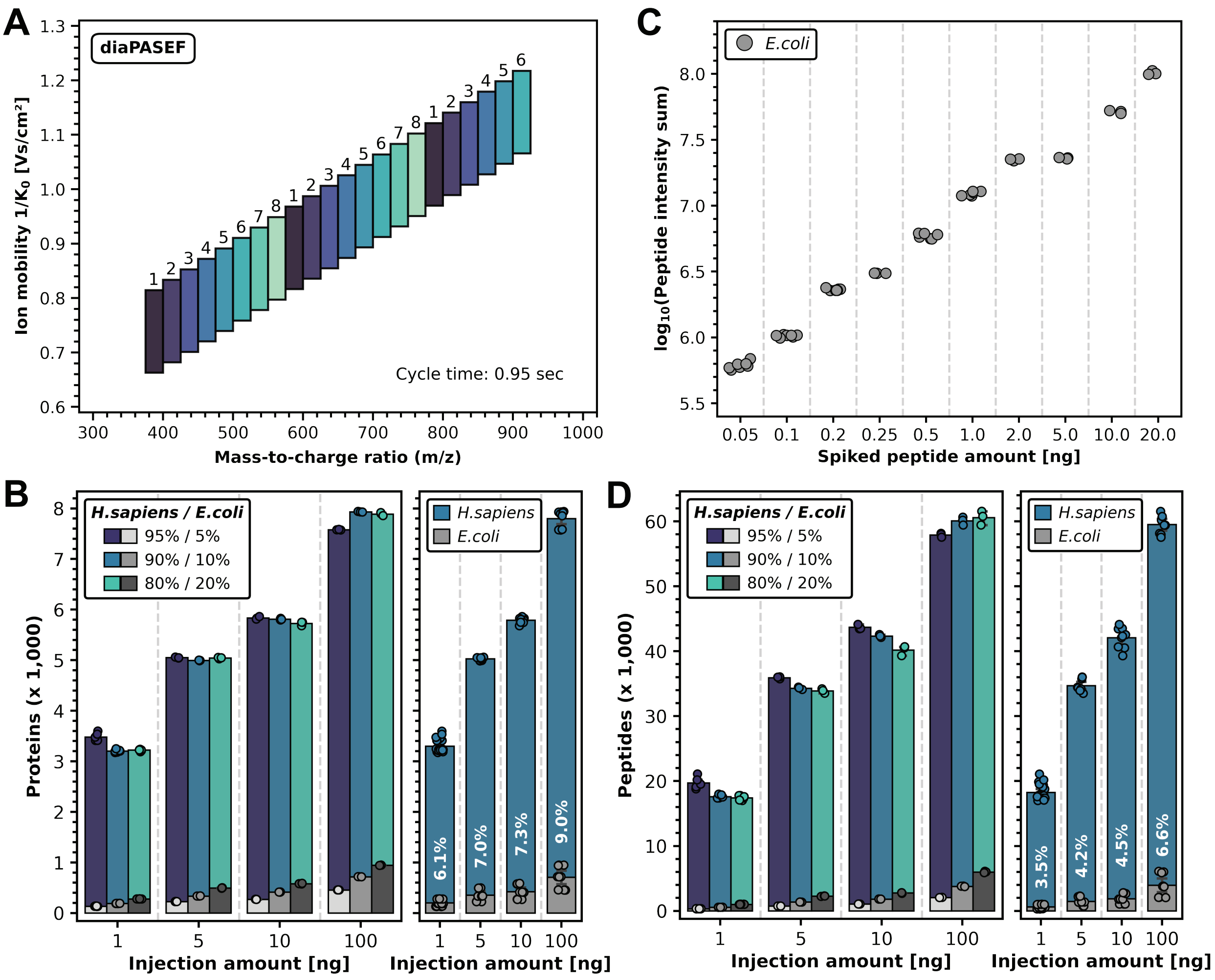

Figure S1: Measurement and assessment of ME samples. (A) Arrangement of diaPASEF windows for the measurement of all samples in this work. In MS2, the method covers an m/z range of 550 Th, ranging from 375 m/z to 925 m/z, and an ion mobility range of about 0.56 1/K0, ranging from 1.22 to 0.66. Windows with equal number are part of the same ramp during precursor fragmentation. (B) Identified *H.sapiens* and *E.coli* protein groups in spiked ME samples (1-ng samples: N = 7; 5 – 100-ng samples: N = 3). Left: Protein groups per injection amount (1 – 100 ng) and *E.coli* spiking ratio (5%, 10% and 20%), respectively. Right: Average protein groups per injection amount (1 – 100 ng) across spiking ratios (1-ng samples: N = 21; 5 – 100-ng samples: N = 9). Proportions of *E.coli* proteins among all identifications are indicated. (C) Cumulative peptide intensities (log_{10}-transformed) per spiked peptide amount, i.e. spiking ratio multiplied with the respective injection amount. (D) Same as (B), but for identified peptides. Source data are provided as a Source Data file.

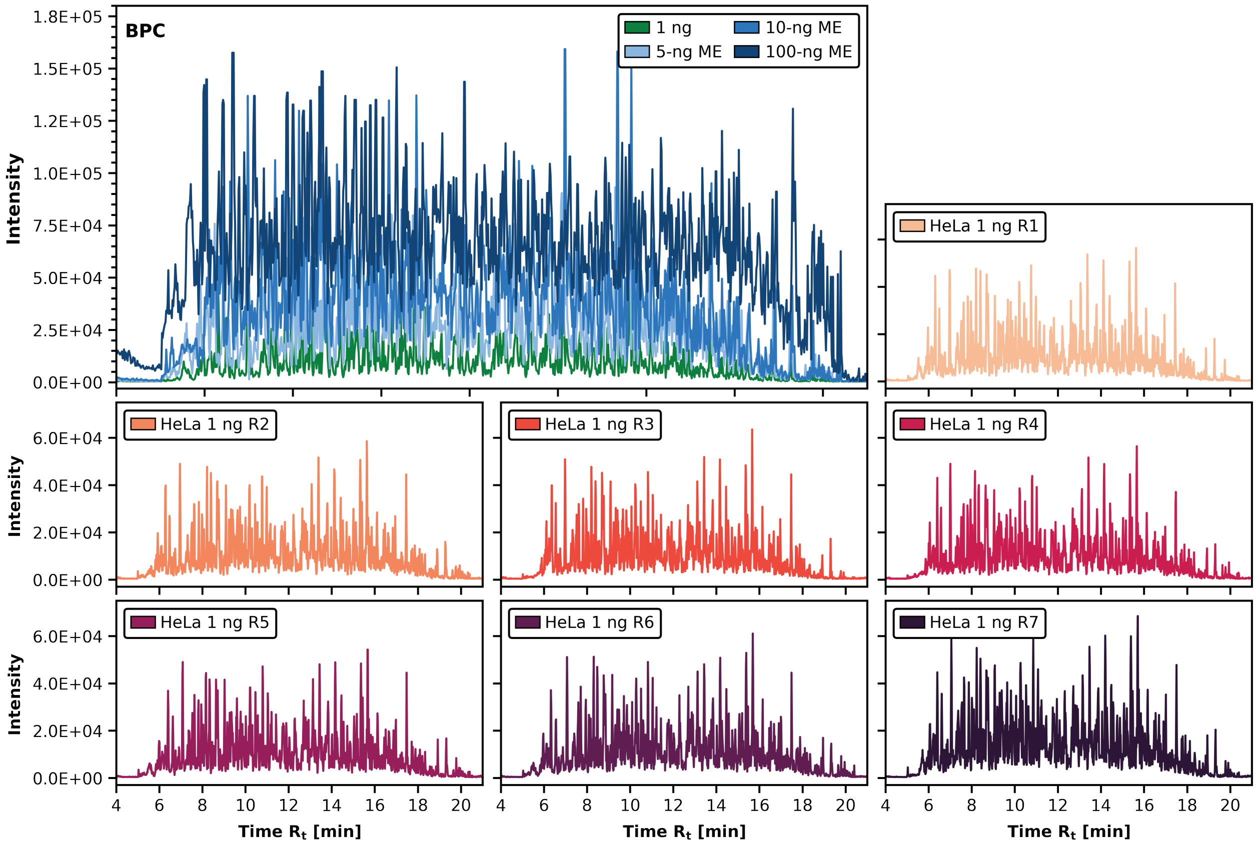

Figure S2: Base peak chromatogram (BPC) of low-input HeLa replicates and MEs. Top panel: overlay of exemplified BPCs of a spiked 5-ng, 10-ng and 100-ng replicate (MEs), respectively, and a non-spiked 1-ng replicate. Bottom panels: BPCs of all seven non-spiked 1-ng replicates. Source data are provided as a Source Data file.

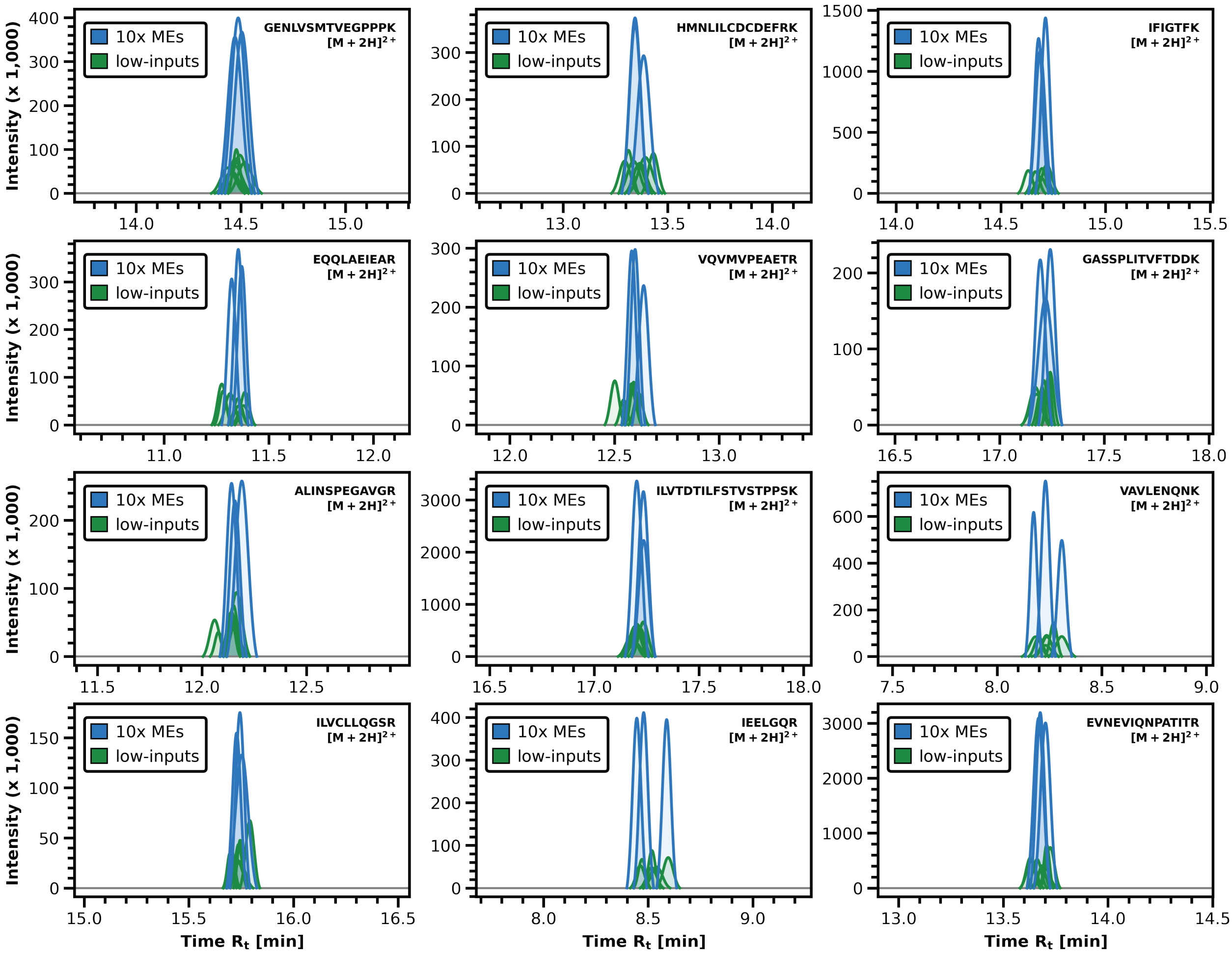

Figure S3: Superimposed elution peaks of selected peptides exclusively identified by DIA-ME analysis. Exemplary peptides from various proteins were identified in 10-ng ME samples (i.e. 10x ME, blue) and they could be retrieved in low-input replicates (1 ng, green), resulting in their identification at full data completeness. Peptide sequences and charge states are indicated. Elution profiles were calculated by Gaussian approximation. Source data are provided as a Source Data file.

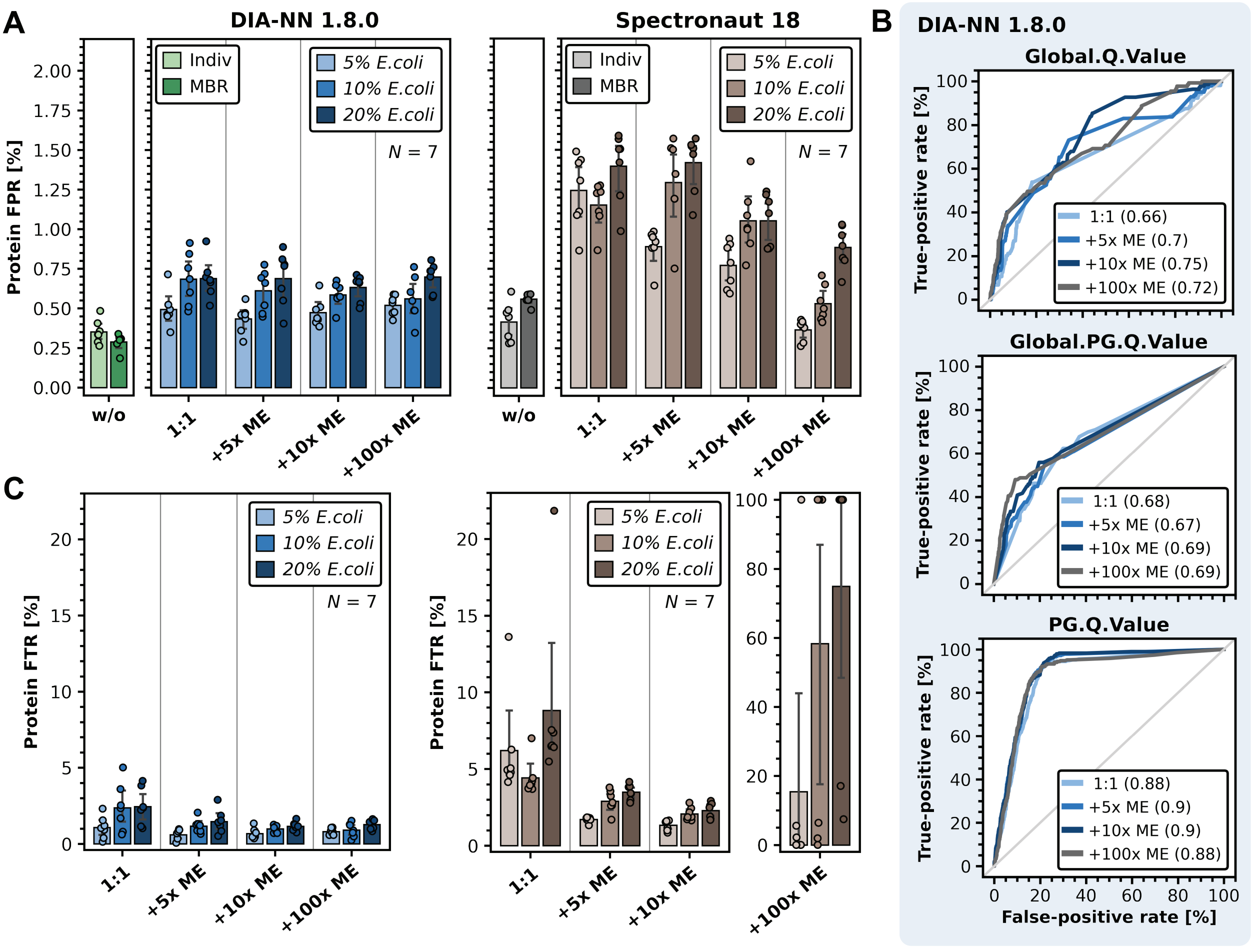

Figure S4: Protein-level false positive and false transfer rates. (A) False positive rate (FPR) of proteins in non-spiked 1-ng *H.sapiens* samples (N = 7) for different types of data analysis and DIA software. Analyses without spiked samples, i.e. without entrapped matching, are indicated in green and grey (light-: without MBR, dark-: MBR), while co-analyses with spiked samples are indicated in blue and brown for DIA-NN and Spectronaut, respectively. The shade of the color indicates the *E.coli* spiking ratio. Error bars are shown as mean ± sd. (B) Receiver operating characteristics (ROC) of non-default q-value filters in DIA-NN for data analyses involving ME samples with 10% spiking ratio (light blue (1:1) to dark grey (100x DIA-ME)). Areas under ROC (AUROC) are indicated in parentheses, while the diagonal line represents a random classification. (C) False transfer rate of proteins, i.e. percentage of *E.coli* proteins among identifications that were transferred by matching, in non-spiked 1-ng *H.sapiens* samples (N = 7) for different types of data analysis and DIA software. Color-coding as in panel A. Error bars are shown as mean ± sd. Source data are provided as a Source Data file.

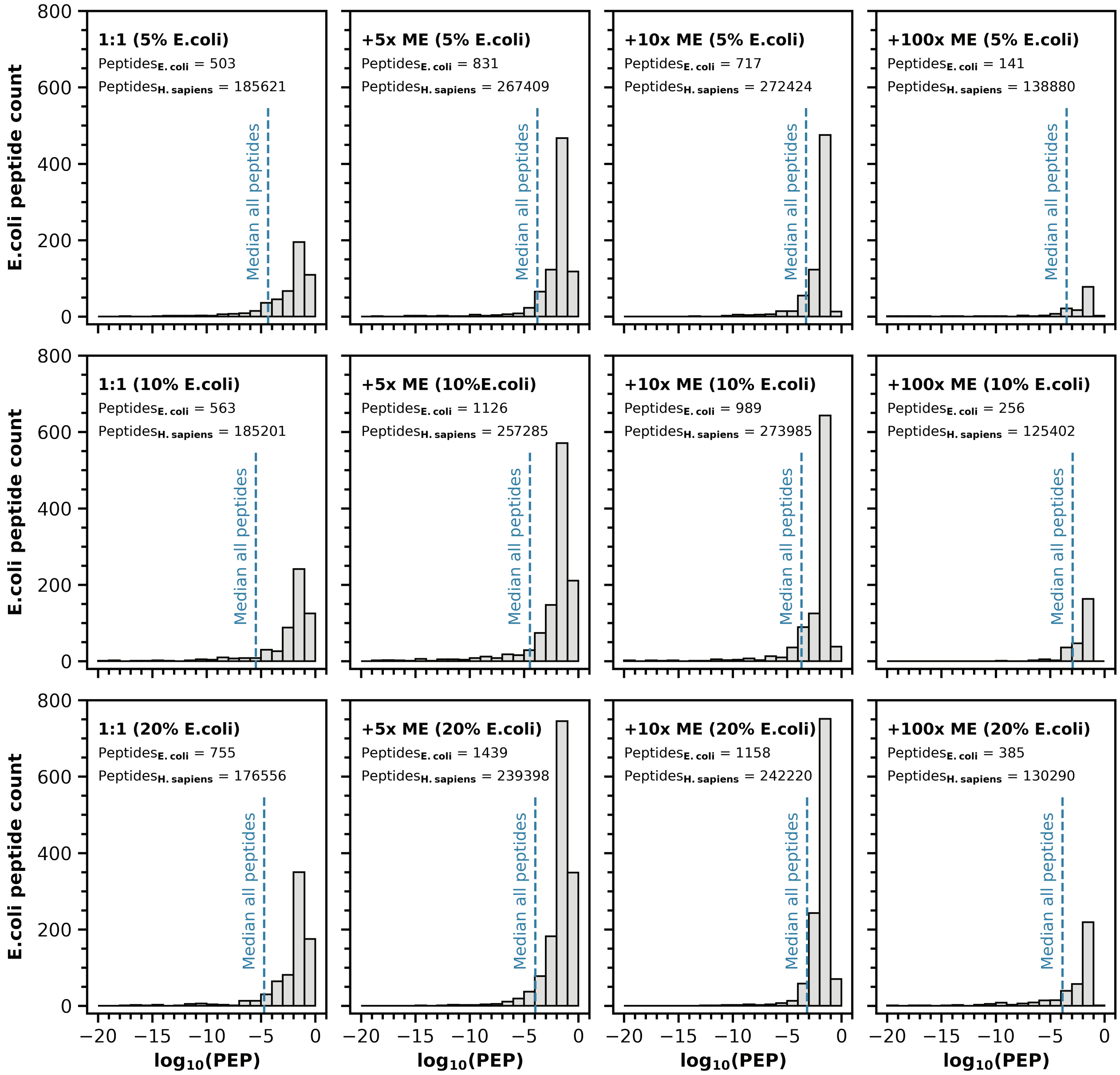

Figure S5: PEP filtering in Spectronaut for low-input DIA data. Histograms of identified *E.coli* peptides in non-spiked 1-ng replicates (N = 7) across their reported posterior error probability (PEP) scores (log_{10}-transformed) after co-analysis with spiked MEs. Number of cumulative *E.coli* and *H.sapiens* peptides, and the observed median PEP scores of all peptides (dashed blue line) are indicated per analysis. Default cutoff in Spectronaut 18 for the peptide-level PEP score is ≤ 0.2. Source data are provided as a Source Data file.

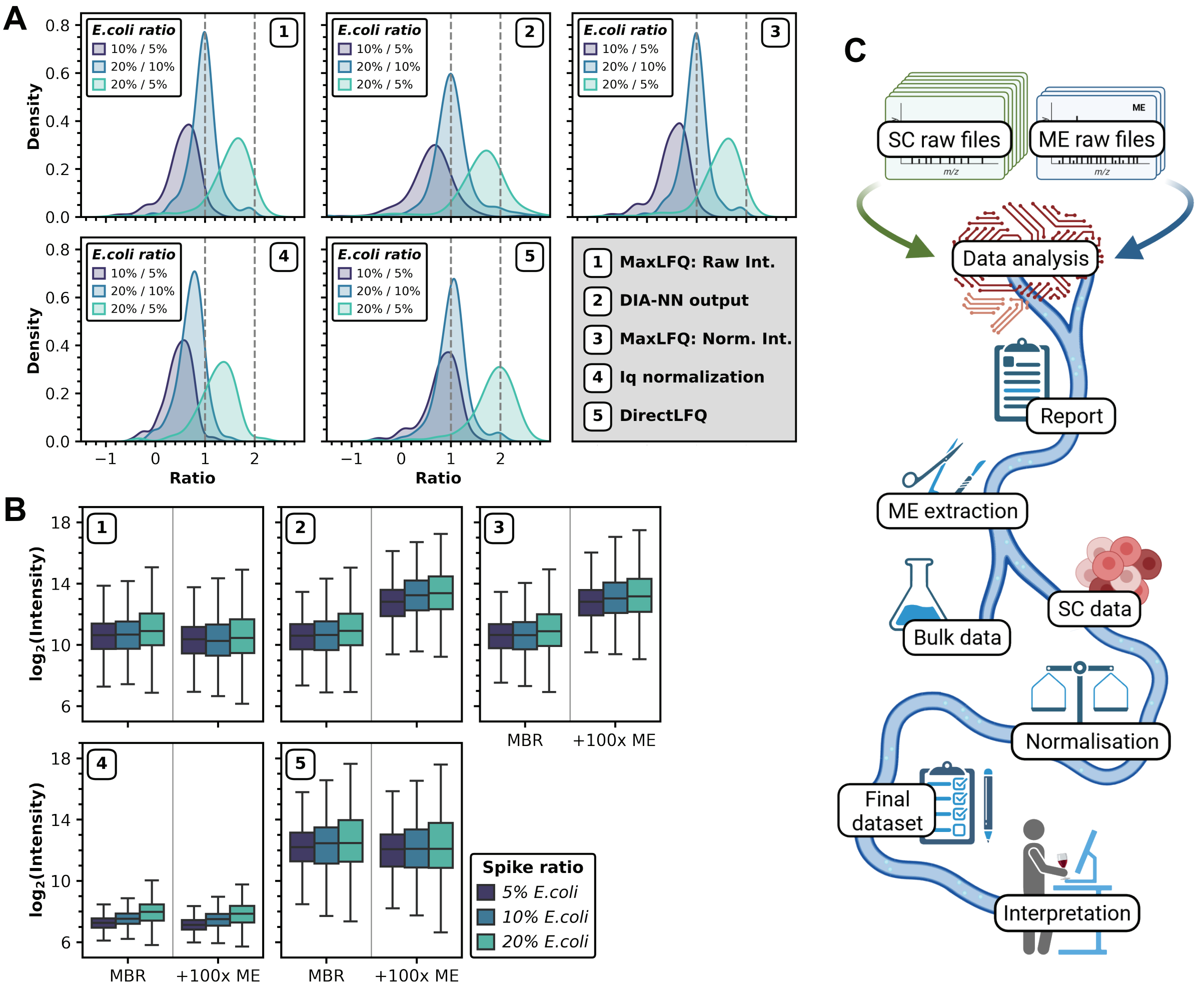

Figure S6: Comparison of different normalization strategies for low-input DIA data analyzed in DIA-NN. (A) Distribution of *E.coli* protein intensity ratios between different spiking amounts after MBR analysis (without MEs) of 1-ng samples (N = 7) using different data normalization strategies. 1: DiaNN R package using raw peptide intensities; 2: protein output matrix of DIA-NN; 3: DiaNN R package using pre-normalized peptide intensities; 4: iq normalization in R; 5: directLFQ normalization in Python. (B) Box plots of reported *E.coli* protein intensities (log_2-transformed) after MBR and 100x DIA-ME analysis of 1-ng samples (N = 7) containing different spiking ratios (5%: dark blue; 10%: blue; 20%: green) and after data normalization strategies of panel A (ME samples were removed from the report file before normalization). Boxes represent the data dispersion between the first and third quartile with the inner line specifying the median of the distribution. Whiskers indicate 1.5-times the inter quartile range. (C) Data pipeline for implementing DIA-ME: low-input samples of interest (e.g. single-cell (SC) data) are searched using a DIA data analysis software together with a small number of MEs, while inter-sample matching by MBR is permitted (settings not shown). The resulting report file contains all identified peptides, including those from ME samples. The latter information is removed (drop respective columns/rows) before subsequent peptide intensity normalization, but can be recycled to serve as a comparative bulk data set. Source data are provided as a Source Data file.

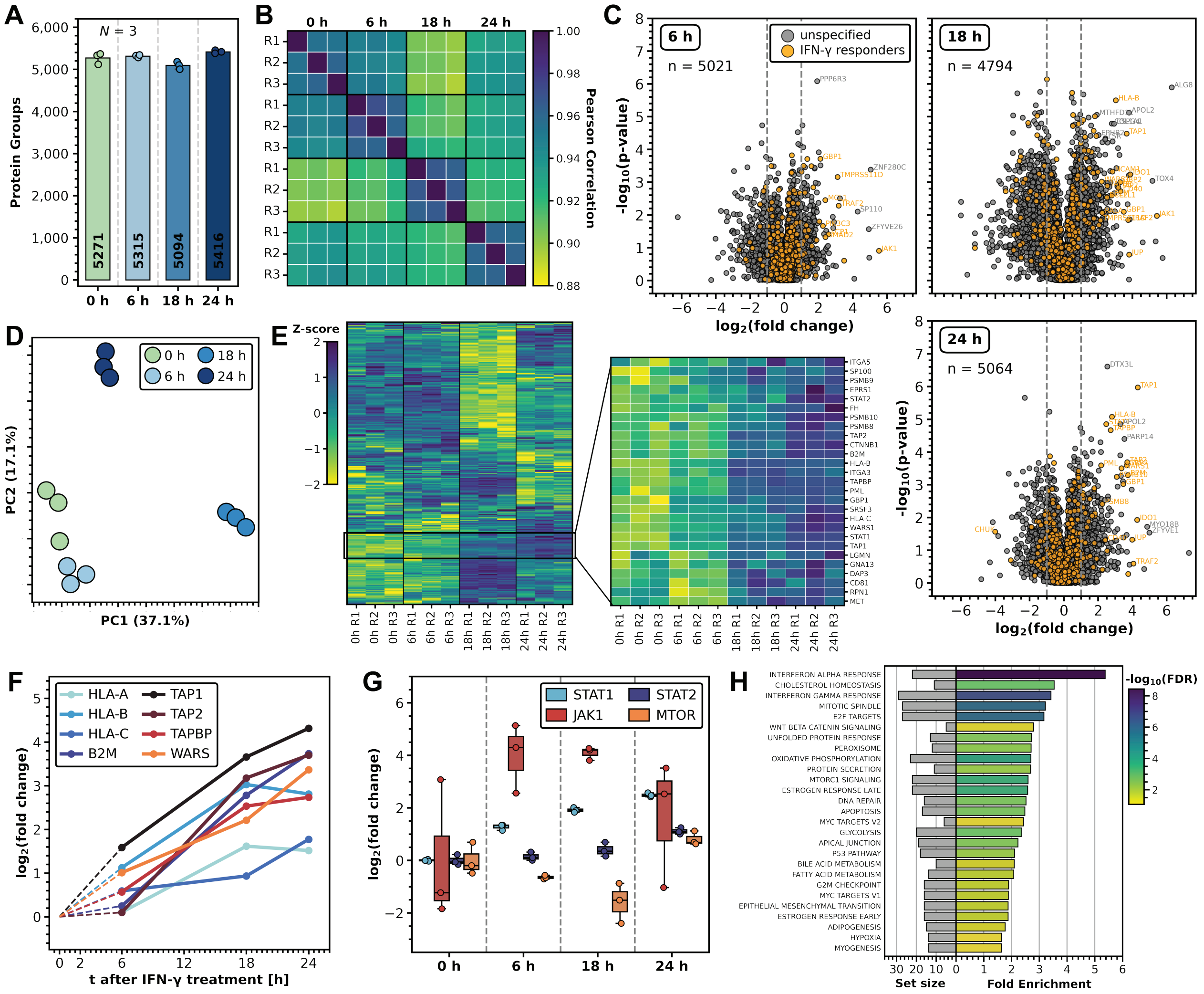

Figure S7: 200-ng bulk analysis of IFN-γ-treated U-2 OS cells. (A) Protein group identifications per time-point replicate (control samples: green, treated samples: blues) (N = 3 individual cell cultures). Error bars are shown as mean ± sd. (B) Pearson correlation heatmap of time-point replicates. (C) Volcano analysis of two-sided Student's t-test results between time-point samples and 0 hours control sample. n: number of differentially expressed proteins. Proteins described to be involved in the IFN-γ-response depited in yellow. Dashed lines indicate a fold change of 2 and -2, respectively. (D) PCA analysis of time-point samples. (E) Heatmap of known IFN-γ-responsive proteins after hierarchical clustering by Euclidean distance. Colors illustrate the quantitative changes compared to the protein's median across all samples by Z-score. The black box outlines an identified cluster that shows gradual increasing Z-score over time, enlarged on the right. (F) Line plot of a selection of proteins, taken from the indicated cluster in panel E, showing their gradual up-regulation over the course of treatment. (G) Box plots of observed fold changes of proteins STAT1, STAT2, JAK1 and mTOR over time. (H) Gene set enrichment analysis of proteins that showed up-regulation after 24 hours ($\log_2$ fold change > 0.58) using MSigDB hallmarks. Bars on the right represent the degree of enrichment, while their colors specify the enrichment's FDR. Bars on the left illustrate the size of the enriched term. Source data are provided as a Source Data file.

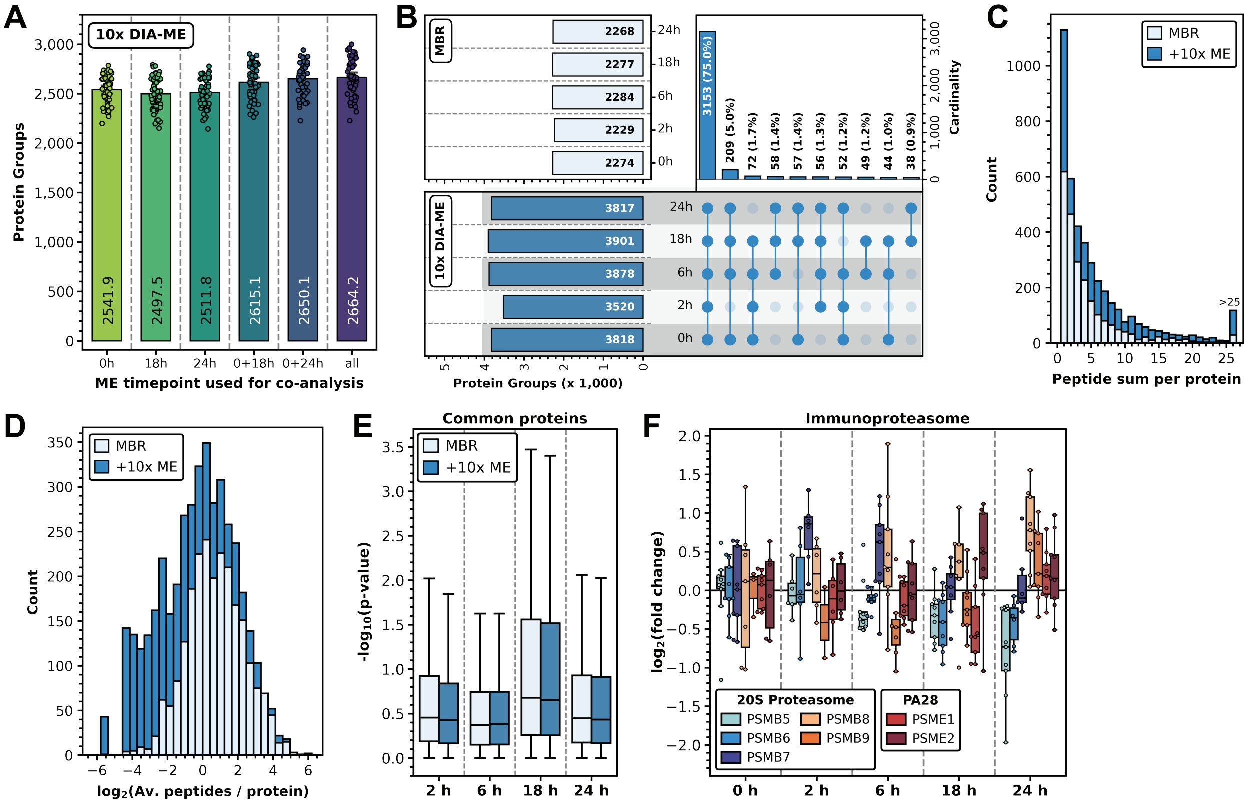

Figure S8: Different additional analyses of 200-pg samples, complementing Fig. 4 and 5. (A) Protein groups in 10x DIA-ME analysis involving different ME time-point samples. (B) UpSet plot of 10x DIA-ME analysis showing total protein group identifications per time-point on the left and protein cardinality, i.e. presence in the time-points, on the right. Numbers and proportions of proteins in the respective time-point combinations, shown by knots, indicated on top of the bars. Total protein groups per time-point after MBR analysis are given as reference. (C) Histogram of total identified peptides per protein for MBR (white) and DIA-ME analysis (blue). (D) Histogram of average peptides per protein and sample (log_2-transformed) for MBR (white) and DIA-ME analysis (blue). (E) P-value distributions from two-sided t-test per time-point and analysis (white: MBR; blue: DIA-ME) as box plot. Boxes show dispersion of values between the first and third quartile with the central line representing the median of the dataset. Whiskers show 1.5-times the inter quartile range. (F) Box plot of protein fold changes (log_2-transformed) of the 20S proteasome and the proteasome activator PA28. Source data are provided as a Source Data file.

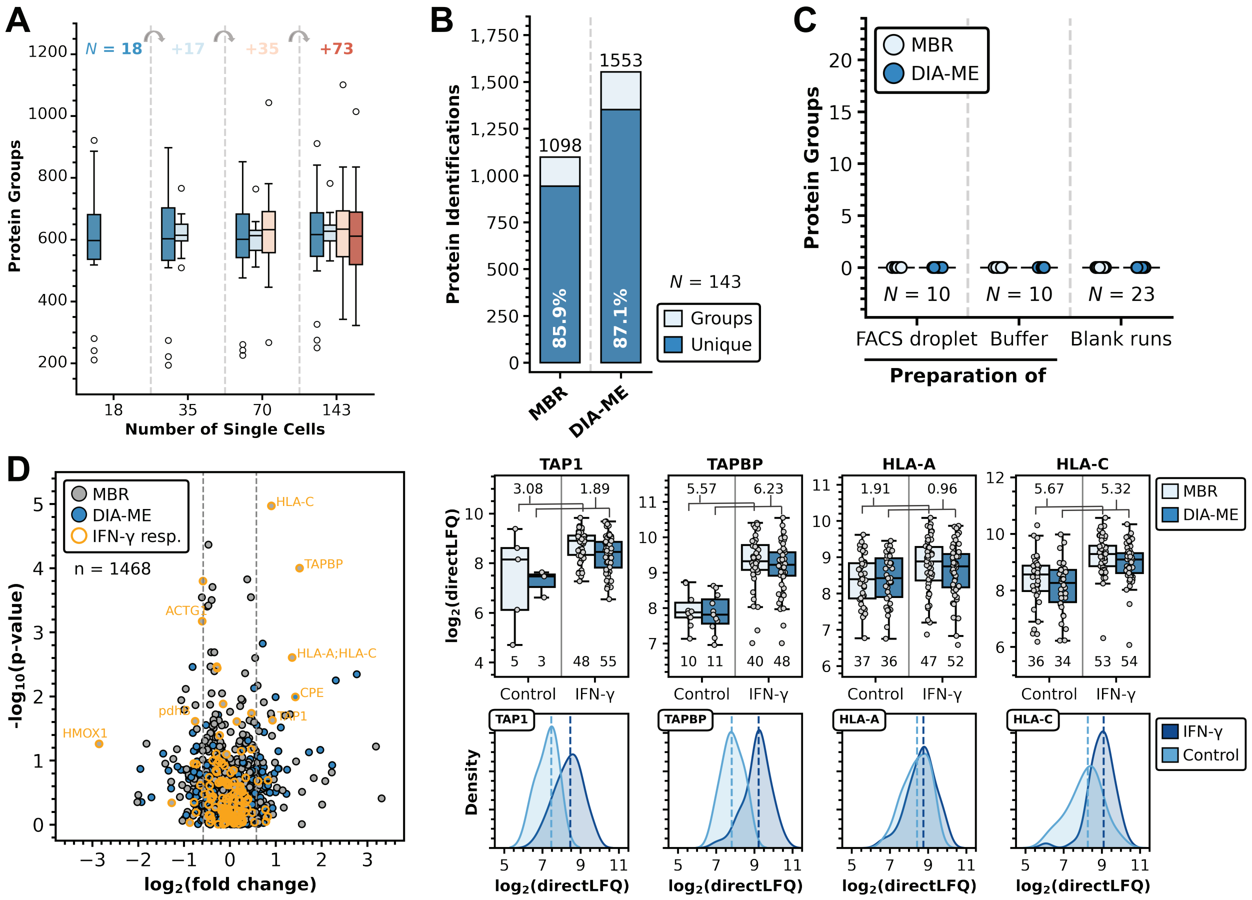

Figure S9: Evaluation of DIA-ME-assisted identification in the analysis of single U-2 OS cells. (A) DIA-ME scalability in the analysis of single cells. Colored box plots represent protein identifications in each group of cells that were successively added to the search, while using a constant number of 10-cell ME samples. (B) Total protein group and unique protein identifications after conventional MBR (white) and DIA-ME analysis (blue) of N = 143 individual cells using 10-cell ME samples. (C) Protein identifications in empty FACS droplets (N = 10) and buffer samples (N = 10) after preparation according to our single-cell workflow, and in LC-blank runs that were measured between single-cell runs in the IFN-γ experiment (N = 23). Identifications shown after MBR (white dots) and DIA-ME analysis (blue). (D) Volcano analysis of two-sided Student's t-test results of IFN-γ-treated and control cells. n: number of differentially expressed proteins. Blue: proteins exclusively observed after DIA-ME analysis; Yellow: protein described to be involved in the cellular response to IFN-γ. (E) Head-to-head evaluation of the four selected proteins (top: box plot; bottom: kernel) for MBR (white) and DIA-ME analysis (blue) and for control (light blue) and IFN-γ treatment (dark blue). Individual protein abundances per cell shown as grey dots (numbers of cells at the bottom). Numbers on the top indicate (negative log_{10}-transformed) p-values from two-sided t-test of control and IFN-γ expressions. Source data are provided as a Source Data file.

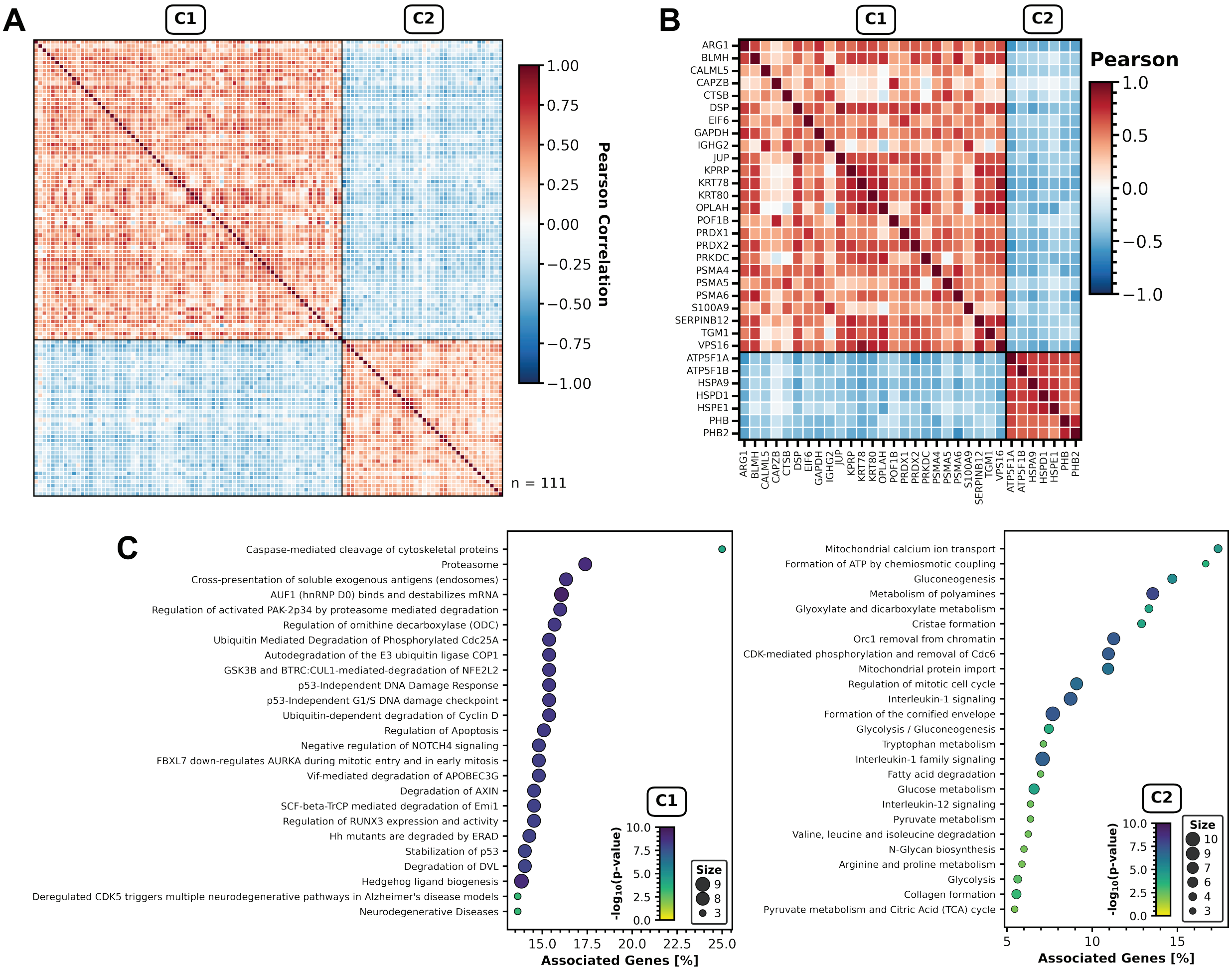

Figure S10: Analysis of Pearson correlation modules, complementing Fig. 7. (A) Enlarged version of the two clusters C1 and C2 from Figure 7A. (B) Exemplified heatmap of highly correlated proteins in clusters C1 and C2 from panel A. (C) Gene set enrichment analysis of clusters C1 and C2 shown as bubble plot. Enriched terms are indicated on the left. Bubble size represents the term size, while the bubble color specifies the enrichment's FDR. Source data are provided as a Source Data file.

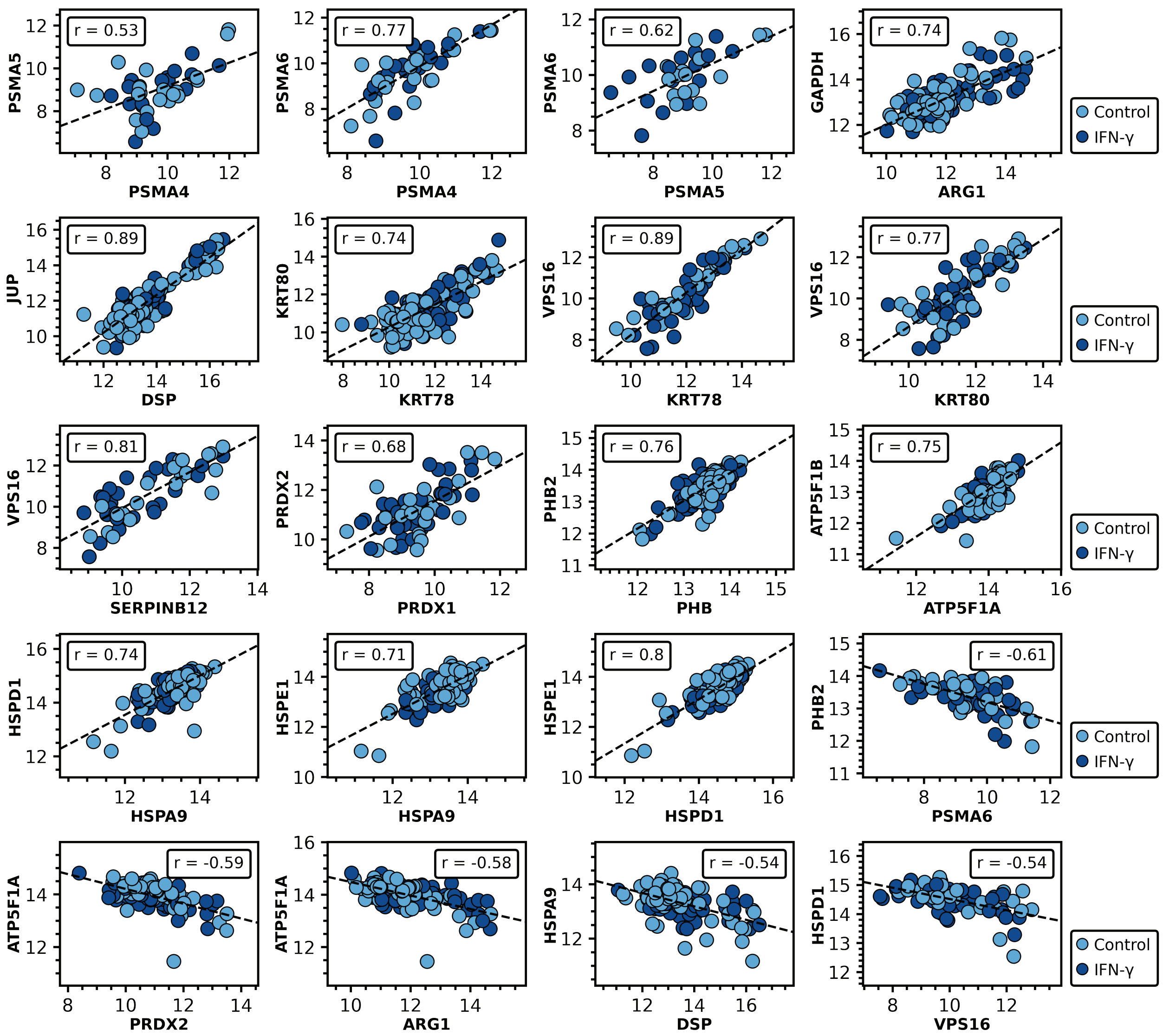

Figure S11: Examples of pairwise-correlated and anti-correlated proteins. Scatter plots of (log_2-transformed) protein intensities observed in individual cells with protein names indicated at the x- and y-axis. Dark blue: IFN-γ-treated cells; blue: control cells. Linear regressions by Pearson are represented as dashed lines with the respective correlation r indicated. Source data are provided as a Source Data file.

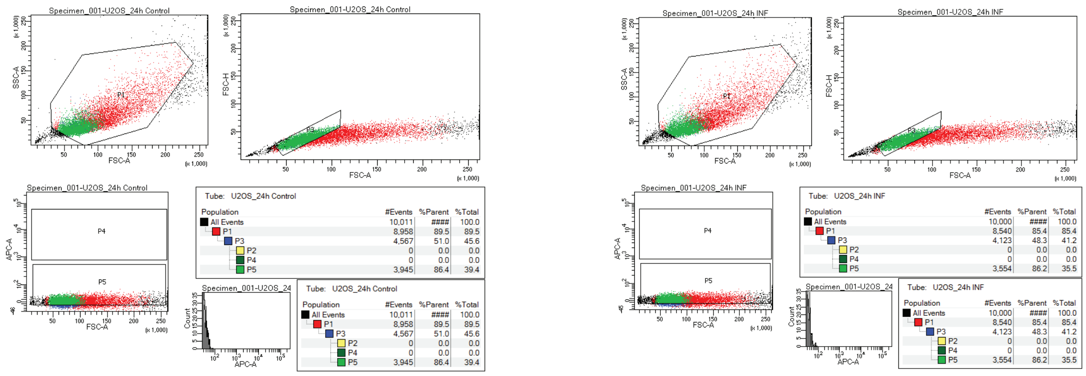

Figure S12. Flow cytometry gating strategy for fluorescence-activated cell sorting (FACS). The gating sequence begins with a forward scatter (FSC) versus side scatter (SSC) plot to identify the main cell population while excluding debris. Next, doublet discrimination is performed using FSC-A versus FSC-H plots. Live cells are selected by gating on a live/dead dye exclusion plot. This gating strategy was used to sort and isolate the single-cell population into individual wells of a plate for downstream analyses. Controls for gating were included as detailed in the Methods section.
