# Supplementary material for: Enhanced feature matching in single-cell proteomics characterizes IFN-γ response and co-existence of cell states
Source: Nat Commun. 2024 Sep 26;15:8262. doi: 10.1038/s41467-024-52605-x (PMC11427561; doi:10.1038/s41467-024-52605-x)
Supplement: Supplementary file 2 — Reporting Summary [file 41467_2024_52605_MOESM2_ESM.pdf]

nature portfolio

Corresponding author(s): Jeroen Krijgsveld

Last updated by author(s): Sep 9, 2024

Reporting Summary

Nature Portfolio wishes to improve the reproducibility of the work that we publish. This form provides structure for consistency and transparency in reporting. For further information on Nature Portfolio policies, see our Editorial Policies and the Editorial Policy Checklist.

Statistics

For all statistical analyses, confirm that the following items are present in the figure legend, table legend, main text, or Methods section.

n/a	Confirmed	
☐	☒	The exact sample size (n) for each experimental group/condition, given as a discrete number and unit of measurement
☐	☒	A statement on whether measurements were taken from distinct samples or whether the same sample was measured repeatedly
☐	☒	The statistical test(s) used AND whether they are one- or two-sided<br>*Only common tests should be described solely by name; describe more complex techniques in the Methods section.*
☐	☒	A description of all covariates tested
☐	☒	A description of any assumptions or corrections, such as tests of normality and adjustment for multiple comparisons
☐	☒	A full description of the statistical parameters including central tendency (e.g. means) or other basic estimates (e.g. regression coefficient) AND variation (e.g. standard deviation) or associated estimates of uncertainty (e.g. confidence intervals)
☐	☒	For null hypothesis testing, the test statistic (e.g. F, t, r) with confidence intervals, effect sizes, degrees of freedom and P value noted<br>*Give P values as exact values whenever suitable.*
☒	☐	For Bayesian analysis, information on the choice of priors and Markov chain Monte Carlo settings
☐	☒	For hierarchical and complex designs, identification of the appropriate level for tests and full reporting of outcomes
☐	☒	Estimates of effect sizes (e.g. Cohen's d, Pearson's r), indicating how they were calculated

Our web collection on statistics for biologists contains articles on many of the points above.

Software and code

Policy information about availability of computer code

Data collection	A timsTOF Pro (Bruker Daltonics) and a Orbitrap Fusion Tribrid mass spectrometer (Thermo Fisher Scientific) were used for data acquisition.
Data analysis	Raw MS data from DDA acquisition methods was processed using Maxquant (2.0.3) and Raw MS data from DIA acquisition methods was processed with DIA-NN (1.8.0) or Spectronaut 18. Further processing of the data was carried out using Python code (version 3.9.12) in the Jupyter Notebook environment (v6.4.8). We employed Pandas (v1.4.2) and NumPy (v1.21.6) packages for data handling, and used the Matplotlib (v3.5.1) and Seaborn (v0.11.2) packages for data plotting. Further statistical calculations, such as two-sided Student's t-tests, hierarchical clustering and Gaussian approximation to model extracted ion chromatograms, were conducted by the SciPy (v1.7.3) package, while ROC curve calculation, dimensional reduction and data imputation were performed using Scikit-learn (v1.0.2). Moreover, we utilized the UpSetPlot package (v0.6.1) for UpSet plotting. For data normalization, we employed the directLFQ package (v0.2.8) after excluding ME samples from the report table and filtering on Q.Value ≤ 0.01, Lib.Q.Value ≤ 0.01 and Lib.PG.Q.Value ≤ 0.01. To evaluate the performance of data normalization, we compared directLFQ to the DiaNN (v1.0.1) and iq (v1.9.6) packages using R Studio while applying the same filter criteria. Gene set enrichment analysis (GSEA) was conducted utilizing the R package clusterProfile (v3.12.0) based on the "fgsea" algorithm. For the assessment of erroneous feature detection in DIA-NN and Spectronaut, we used a number of equations that are indicated under the "data analysis" statement in the method section.<br>All relevant code used for the analysis of data in this work is stored in a GitHub repository that can be accessed via https://github.com/krijgsveld-lab/DIA-ME.

For manuscripts utilizing custom algorithms or software that are central to the research but not yet described in published literature, software must be made available to editors and reviewers. We strongly encourage code deposition in a community repository (e.g. GitHub). See the Nature Portfolio guidelines for submitting code & software for further information.

Data

Policy information about availability of data

All manuscripts must include a data availability statement. This statement should provide the following information, where applicable:

- Accession codes, unique identifiers, or web links for publicly available datasets
- A description of any restrictions on data availability
- For clinical datasets or third party data, please ensure that the statement adheres to our policy

The acquired raw LC-MS/MS data and processed report files generated in this study have been deposited in the ProteomeXchange Consortium via the PRIDE partner repository under the accession codes PXD053462 [https://www.ebi.ac.uk/pride/archive/projects/PXD053462] (E.coli-spiked experiment), PXD048162 [https://www.ebi.ac.uk/pride/archive/projects/PXD048162] (bulk IFN-γ experiment), PXD053473 [https://www.ebi.ac.uk/pride/archive/projects/PXD053473] (low-input IFN-γ experiment) and PXD053464 [https://www.ebi.ac.uk/pride/archive/projects/PXD053464] (single-cell experiment). In addition, we uploaded SDRF metadata with our evaluation data set (E.coli-spiked experiment). All data generated in this study are provided in the Supplementary Information and in the Source Data file.

Research involving human participants, their data, or biological material

Policy information about studies with human participants or human data. See also policy information about sex, gender (identity/presentation), and sexual orientation and race, ethnicity and racism.

Reporting on sex and gender	n.a.
Reporting on race, ethnicity, or other socially relevant groupings	n.a.
Population characteristics	n.a.
Recruitment	n.a.
Ethics oversight	n.a.

Note that full information on the approval of the study protocol must also be provided in the manuscript.

Field-specific reporting

Please select the one below that is the best fit for your research. If you are not sure, read the appropriate sections before making your selection.

[x] Life sciences [ ] Behavioural & social sciences [ ] Ecological, evolutionary & environmental sciences

For a reference copy of the document with all sections, see nature.com/documents/nr-reporting-summary-flat.pdf

Life sciences study design

All studies must disclose on these points even when the disclosure is negative.

Sample size	To provide strong statistical evidence, we used seven technical replicates for our method evaluation (E.coli-spike experiment), and three replicates of 5-ng, 10-ng and 100-ng samples as well as seven replicates of 1-ng samples with three different E.coli-spiking ratios, respectively, as matching enhancers (MEs) for the DIA-ME analysis (total of 55 injections). For our bulk IFN-γ experiment (200 ng), we used three biological replicates (independent cell cultures) per time-point (4) (total of 12 injections). For our low-input IFN-γ experiment (200 pg), we used three biological replicates (independent cell cultures), each injected in three technical replicates, per time-point (6), and three biological replicates (independent cell cultures) per time-point (6) as ME samples (total of 108 injections). For the single-cell experiment, we used 143 individual cells and fifteen 10-cell samples as MEs (total of 158 injections). Hence, the total number of injections in this work was 333.
Data exclusions	We excluded one biological replicate of the 2-hours time-point in the low-input IFN-γ treatment experiment (200 pg) due to problems in the data acquisition resulting in poor quantification data.
Replication	All data replications were successful except for replicates mentioned under "data exclusions"
Randomization	Samples from all experiments were measured in a randomized order. Single control and treated cells were measured in an alternate order.
Blinding	Not applicable given the in-vitro and technical focus of the study.

Reporting for specific materials, systems and methods

We require information from authors about some types of materials, experimental systems and methods used in many studies. Here, indicate whether each material, system or method listed is relevant to your study. If you are not sure if a list item applies to your research, read the appropriate section before selecting a response.

Materials & experimental systems

n/a	Involved in the study
[x]	[ ] Antibodies
[ ]	[x] Eukaryotic cell lines
[x]	[ ] Palaeontology and archaeology
[x]	[ ] Animals and other organisms
[x]	[ ] Clinical data
[x]	[ ] Dual use research of concern
[x]	[ ] Plants

Methods

n/a	Involved in the study
[x]	[ ] ChIP-seq
[ ]	[x] Flow cytometry
[x]	[ ] MRI-based neuroimaging

Eukaryotic cell lines

Policy information about cell lines and Sex and Gender in Research

Cell line source(s)	U-2 OS cells were obtained from ATCC (HTB-96)
Authentication	Cell line was not authenticated
Mycoplasma contamination	Cell line was tested for Mycoplasma contamination.
Commonly misidentified lines (See ICLAC register)	To the best of our knowledge no misidentified cell lines have been used in this study.

Plants

Seed stocks	n.a.
Novel plant genotypes	n.a.
Authentication	n.a.

Flow Cytometry

Plots

Confirm that:

- [ ] The axis labels state the marker and fluorochrome used (e.g. CD4-FITC).
- [ ] The axis scales are clearly visible. Include numbers along axes only for bottom left plot of group (a 'group' is an analysis of identical markers).
- [ ] All plots are contour plots with outliers or pseudocolor plots.
- [ ] A numerical value for number of cells or percentage (with statistics) is provided.

Methodology

Sample preparation	Cells were prepared by gentle trypsin digestion (0.25%) to establish a homogeneous population of singularized cells. Approximately one million cells were diluted in 1.5 mL PBS (Sigma-Aldrich) before FACS sorting.
Instrument	BD FACSAria III instrument (BD Biosciences)
Software	*Describe the software used to collect and analyze the flow cytometry data. For custom code that has been deposited into a community repository, provide accession details.*
Cell population abundance	Cells were sorted to obtain single cells and 10-cell samples per well of a 384-well plate
Gating strategy	Default settings for optical filters and mirrors were employed to facilitate the detection of the scattered signals.

- [x] Tick this box to confirm that a figure exemplifying the gating strategy is provided in the Supplementary Information.
