# Supplementary material for: Enhanced feature matching in single-cell proteomics characterizes IFN-γ response and co-existence of cell states
Source: Nat Commun. 2024 Sep 26;15:8262. doi: 10.1038/s41467-024-52605-x (PMC11427561; doi:10.1038/s41467-024-52605-x)
Supplement: Supplementary file 4 — Description of Additional Supplementary Files [file 41467_2024_52605_MOESM4_ESM.pdf]

File Name: **Supplementary_Data_1_Software_Configuration**

Description: How raw files were combined for all data searches performed in DIA-NN and their respective software configuration

File Name: **Supplementary_Data_2_Ecoli_DIA-NN**

Description: Protein output intensities of DIA-NN searches in the E.coli-spiked experiment; related to Figure 1 – 3

File Name: **Supplementary_Data_3_Ecoli_Spectronaut**

Description: Protein output intensities of Spectronaut searches in the E.coli-spiked experiment; related to Figure 1 – 3

File Name: **Supplementary_Data_4_Ecoli_DIA-NN_directLFQ_Accuracy**

Description: DirectLFQ-normalized protein intensities of DIA-NN searches performed to determine the quantitative accuracy in low-input data; related to Figure 3E

File Name: **Supplementary_Data_5_Bulk_IFNy-200ng**

Description: MaxLFQ-normalized protein intensities of MaxQuant searches (not yet median normalized) in the 200-ng time series experiment of IFN-γ treated U-2 OS cells on a Fusion Orbitrap; Results of two-sided Student's t-tests; related to Supplementary Figure 7

File Name: **Supplementary_Data_6_IFNy-200pg_directLFQ**

Description: DirectLFQ-normalized protein intensities from the analysis of 200-pg time-point samples after IFN-γ treatment in DIA-NN; results of DIA-ME analysis shown for using ME samples from the control (0 hours) and 24 hours treatment condition; Results of two-sided Student's t-tests; related to Figure 4 & 5

File Name: **Supplementary_Data_7_IFNy_database**

Description: Database of STRING- and Gene Ontology-derived proteins (gene symbols and accessions) described to be involved in the cellular response to IFN-γ

File Name: **Supplementary_Data_8_IFNy_single-cells_directLFQ**

Description: DirectLFQ-normalized protein intensities from the analysis of single U-2 OS cells in DIA-NN; DIA-ME analysis involved 10-cell ME samples; Results of two-sided Student's t-test, related to Figure 6

File Name: **Supplementary_Data_9_Single-Cell_ProteinCo-Expression**

Description: Pearson correlations of protein co-expression analysis among single U-2 OS cells; related to Figure 7
